# Behavioural Profile of Agility Dogs Using the Mini‐CBARQ in Santiago de Chile

**DOI:** 10.1155/vmi/4712339

**Published:** 2026-11-01

**Authors:** Sofia Torres, Paola Mujica, Hernan Cañon Jones

**Affiliations:** ^1^ Facultad de Medicina Veterinaria y Agronomia Universidad de Las Americas Santiago Chile

**Keywords:** agility, behaviour, dog, Mini-CBARQ, welfare

## Abstract

**Objectives:**

To evaluate behavioural traits and overall well‐being in agility dogs in Chile using a standardised questionnaire and to explore how agility participation relates to behaviour patterns such as attachment, trainability and anxiety.

**Methods:**

A cross‐sectional pilot survey of 10 dog guardians participating in agility competitions in Santiago, Chile, was conducted. Inclusion criteria required adult guardians and clinically healthy dogs aged 1–8 years with recent veterinary examinations. Behavioural assessment was performed using the Mini‐CBARQ, covering aggression, fear, attachment and trainability. Data were analysed descriptively, and associations between traits were explored using Spearman correlations.

**Results:**

A distinct behavioural profile was observed. High levels of attachment and attention‐seeking behaviours (72.5%) and trainability (70.8%) were observed. Low levels of aggression (9.8%) and separation‐related behaviours (8.3%) were reported overall. A positive correlation was identified between excitability and separation‐related behaviours, suggesting that more excitable dogs may be more prone to separation difficulties. Most dogs were Border Collies (90%) with a mean age of 4.05 years.

**Clinical Significance:**

Agility participation is associated with strong dog‐handler bonding and high trainability, which may support obedience and performance in clinical and behavioural contexts. However, increased attachment and excitability may predispose some dogs to separation‐related issues, highlighting the need for balanced behavioural management and routine welfare monitoring in active canine patients.

## 1. Introduction

The domestic dog (*Canis lupus familiaris*) is a species of significant interest in behavioural studies because it has been undergoing high selective pressures over thousands of years, but similarities in personality traits to wolves (*Canis lupus*) persist [1]. Dogs are renowned for their exceptional ability to interact with humans [2]. This connection dates to ancestral times, encompassing an association spanning over a thousand years [3]. Dogs excel in social cognition tasks, primarily attributed to their observational skills [4], interpreting visual, auditory and gestural signals [5]. They can observe humans for information or problem‐solving [4] and can perceive human emotional states [5]. Similarly, humans possess the ability to understand signals emitted by dogs, facilitating effective communication [6]. Dog owners need to develop nonverbal communication skills to prepare dogs for various occupational roles such as defence, military service, hunting, therapy and canine sports (including obedience, agility and frisbee sports), skills crucial for daily obedience [6]. Historically, dogs have been trained for various functions, ranging from traditional roles such as hunting, herding and guarding to participating in modern canine sports such as agility [7]. This sport has rapidly gained global popularity [8]. Agility competitions promote positive training and aim to show obedience and physical fitness [3]. Owners of these dogs dedicate considerable time and effort to daily training, which is reflected in their success in sports competitions [9]. Certain breeds, such as Border Collies and Pyrenean Shepherds, are particularly well‐suited to this sport due to their high energy levels and willingness to cooperate with humans [10]. However, breeds with more docile temperaments participate in agility and are especially popular among older individuals who may have reduced physical fitness and stamina [9].

A variety of methods and tools have been employed to evaluate canine personalities and temperaments, reflecting the multidimensional nature of this assessment. Studies have used owner‐completed questionnaires to assess traits such as dominance, aggression and sociability, shedding light on the importance of understanding human perspectives in evaluating canine personalities [11, 12]. Behaviour coding and free‐choice profiling have also been employed to capture canine personality, allowing observers to use their vocabulary to rate and compare dog behaviours [13]. In addition, portable technologies such as accelerometers have been explored to infer canine personality, demonstrating the potential of innovative approaches for evaluating canine traits [14]. The Canine Behavioural Assessment and Research Questionnaire (C‐BARQ) is a widely used tool for evaluating various aspects of canine behaviour [15]. It has been employed to compare behaviour across different dog populations, study behavioural differences among breeds and predict training outcomes in juvenile guide dogs [16–18]. The C‐BARQ has also been used to characterise the behaviours of dogs surrendered to shelters and identify suitable guide or service dogs as early as 6 months of age [16, 19]. In addition, the questionnaire has been adapted to measure impulsivity and ADHD‐related behaviours in dogs, reflecting its versatility in capturing a wide range of behavioural traits [20]. More recently, a shortened version has been developed, called Mini‐C‐BARQ, which has been validated using dogs of different ages [21, 22].

This pilot study aimed to evaluate the behavioural profile and overall welfare indicators of agility dogs in Chile using the Mini‐CBARQ, while exploring the relationships between behavioural traits, agility participation and guardian sociodemographic characteristics.

## 2. Materials and Methods

A total of 10 dog owners with their dogs actively competing in the last three months in agility tournaments were surveyed. This sample size achieved 29% of agility dogs in Santiago de Chile, based on the current Agility dogs registered in Agility Chile (35 total dogs in Santiago), an ONG dedicated to promoting and organising competitions. Additional inclusion criteria included the following: owners aged 18 or over, clinically healthy dogs aged 1–8 years (cut‐offs are related to typical adult dogs [23], excluding young or senior dogs), who had undergone a veterinary check‐up within the last 12 months. Dogs of any breed were eligible.

The Mini‐C‐BARQ questionnaire [24] was employed as the primary instrument. In brief, the questionnaire includes items assessing behaviours such as aggression directed towards humans (owner and strangers), fear of dogs and people, separation‐related behaviours, attention‐seeking and attachment, training responsiveness, excitability, contact sensitivity, energy levels and other behavioural traits. Responses were recorded using a Likert scale in 7 behavioural categories (excitability, aggression, fear and anxiety, separation anxiety, attachment and attention‐seeking, trainability and others) and interpreted as the higher the score, the higher the behavioural trait.

We also measured dog welfare using closed questions based on the current Responsible Tenure of Dogs law applicable in Chile, such as up‐to‐date vaccinations and antiparasitic drugs, regular full veterinary check‐ups, registration in the National Dog Registry, microchipping, availability of food/water and living space. In addition, sociodemographic information about the owner was collected, including gender, age, educational level, housing type and the presence and number of other dogs or cats. The survey was designed to be used online utilising Microsoft Forms, and raw data were exported into Microsoft Excel spreadsheets for subsequent analysis. Ethical approval was waived by the Scientific Ethics Committee of UDLA as only nonessential questions were obtained from owners, and no animal manipulation, contact or intervention was carried out. All owners had to read and sign an informed consent electronically, which explained the objectives of the study.

Descriptive statistics were calculated for all variables. Nonparametric Pearson’s chi‐square (*X* ^2^) goodness‐of‐fit tests were used to evaluate categorical sociodemographic variables (e.g., guardian gender, education level, dog breed distribution and family member status) against expected uniform distributions. Bivariate associations between Mini‐CBARQ behavioural subscales were tested using Spearman rank correlations (*ρ*). All statistical analyses were conducted in Jamovi (version 2.3) with statistical significance set at *p* = 0.05.

## 3. Results

Most respondents were women (9 vs. 1, 90%, *X* ^2^ = 6.4, df = 1, *p* = 0.01) with an age of 35 ± 9.9 years owing 3.9 ± 2.6 dogs and 1.0 ± 2 0.0 cats. The age of dogs was 4.05 ± 3.2 years, and respondents acquired their dogs at the age of 2.7 ± 0.6 months. Notably, all dogs were purchased. No significant differences were observed in the educational level of respondents, although all individuals had at least completed secondary education (*X* ^2^ = 2.0, df = 3, *p* = 0.567). Regarding pet demographics, 50% of the dogs were female and 50% male, and all dogs (100%) were gonadectomised. Most of the dogs were Border Collies (9 vs. 1, 90%, *X* ^2^ = 6.4, df = 1, *p* = 0.01). There were good welfare and responsible dog guardianship, with 100% of the dogs microchipped, having visited a veterinarian in the past year and being vaccinated with all compulsory vaccines given (*p* < 0.05). This reflects a compliance rate of 98% ± 0.6% with Chilean pet care standards. Notably, 70% of respondents considered their dog a member of the family (7 vs. 3, 70%, *X* ^2^ = 6.2, df = 2, *p* = 0.045).

The relative frequencies obtained for each dimension of the Mini‐CBARQ assessment can be seen in Table 1 and Figure 1, with the highest scores corresponding to behaviours associated with attachment/attention‐seeking, training/obedience and excitability (72.5%, 70.8% and 45%, respectively).

**TABLE 1:** Mini‐CBARQ behavioural scores expressed as normalised mean percentages (% ± SD) across seven behavioural dimensions in agility dogs (*n* = 10).

Dimension	Mean score (%)	Standard deviation (SD)	Range (%)	
Excitability	45.00	0.2648	12.50	7.50
Aggression	9.75	0.0961	0.00	27.50
Fear and anxiety	20.56	0.1647	0.00	58.30
Separation anxiety	8.33	0.0962	0.00	25.00
Attachment and attention‐seeking	72.50	0.3162	0.00	100.00
Trainability	70.83	0.1375	50.0	91.70
Other	22.50	0.1565	0.00	50.00

**FIGURE 1:**
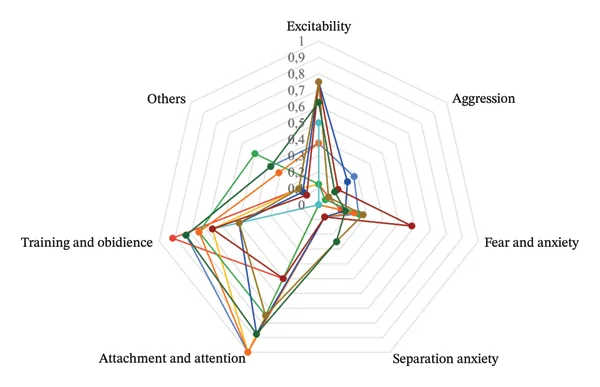
Radar graphics representing each dog according to the dimensions of Mini‐CBARQ. Each colour represents an individual animal, and the farther from the centre, the higher the score for that dimension.

Separation anxiety behaviours and excitability dimensions were positively correlated (Pearson *r* = 0.681, *p* = 0.03). No statistically significant correlation was found between training and excitability dimensions (Pearson *r* = −0.604, *p* = 0.06).

Spearman rank correlation analyses revealed no statistically significant associations between Mini‐CBARQ behavioural traits and guardian sociodemographic variables, including guardian age (*ρ* = 0.12, *p* = 0.74), number of dogs in the household (*ρ* = −0.08, *p* = 0.82) or dog age (*ρ* = 0.15, *p* = 0.68).

## 4. Discussion

Our results showed a distinct behavioural profile in agility dogs, with dogs highly attached to and seeking attention from their owners, and also highly trainable and excitable. In fact, excitability was 45%, which contrasts with the study of behaviour problems in dogs attending clinics in Peru reporting 93.5% [25]. Our pilot study found a strong positive correlation between excitability and separation‐related dimensions, suggesting that an increase in one is associated with an increase in the other. The observed positive correlation between excitability and separation‐related behaviours highlights a key underlying psychometric overlap between these Mini‐CBARQ constructs. While evaluated as separate behavioural domains, both traits are mediated by shared neurobiological substrates involving autonomic arousal and hyperreactivity to environmental or social cues. In sports dogs, high arousal states cultivated during agility training may generalise to nonsporting contexts, lowering the threshold for distress during separation and manifesting as heightened behavioural reactivity when isolated from the handler. The moderate but significant level of excitability in this study may be attributed to the constant stimulation involved in agility sports. Agility is a highly dynamic activity requiring dogs to overcome obstacles within a short timeframe. In addition, dogs in this sport are often motivated by rewards such as food, toys or affection from their guardians, which can heighten excitability during training or competition. Environmental conditions, including noise, the presence of other dogs and crowd size, may also influence these outcomes. This pilot study found low general aggression, which contrasts with another study in Chile which reported different figures for specific aggression types: 56.7% towards strangers, 25.6% towards owners, 45.5% towards unfamiliar dogs and 57.4% towards familiar dogs [26]. Also, in the general population for dogs in northern Mexico [27], we found higher aggressiveness (50%–52%) compared to our pilot study. However, dogs attending veterinary clinics in Peru had a lower level (9.6%) of aggression towards other dogs and 6.1% towards familiar dogs [25]. Although aggression is a primary reason for behavioural consultations due to the significant concern of owners [28], this was not the case in our study. We did not ask how dogs in our pilot study were trained, but there is evidence that agility dogs are trained to follow commands, concentrate and work closely with their handlers, fostering positive behaviours and reducing aggression. Another factor could be the selection of temperamentally suitable dogs for agility sports. Lastly, the dogs’ focus on completing obstacles efficiently may also contribute to lower aggression levels [29, 30].

A total of 20.6% of participants in our pilot study indicated that their dogs displayed fear and anxiety issues. In comparison, Torres [26] reported 37.8% fear of strangers and 41.1% nonsocial fear, such as fear of objects or unfamiliar situations. Dogs may exhibit anxiety or fear when exposed to specific sounds, objects, people or situations. Mild to moderate fear typically manifests as avoidance behaviours, low posture, whining, trembling or agitation. Extreme fear may result in vigorous attempts to escape or hide [31]. Although we did not ask about the type of reinforcement used during training of dogs, the relatively low levels of fear in our study may be influenced by positive reinforcement, which builds confidence and reduces fear in dogs as suggested by other studies [32, 33].

This pilot study found that only 8.3% of dogs experienced separation‐related issues. Torres [26] reported a higher figure of 41.1%, while García Artica [25] presented similar results at 5.8%. The lower percentage in this study may be attributed to controlled exposure to brief separation during training, helping dogs adapt to situations where they are separated from their handlers for short periods. In this pilot study, 72.5% of dogs exhibited attachment and attention‐seeking behaviours, higher than the 53.3% reported by Torres [26] but lower than the 84.2% reported by García Artica [25]. Agility sports require close cooperation between dogs and their handlers. Successfully navigating obstacles necessitates constant attention to cues, fostering a strong bond that may also manifest as excessive vigilance by the guardian. This can contribute to a state of constant anxiety, potentially resulting in separation anxiety. The most debated aspect of separation anxiety is the potential impact of excessive attachment to guardians. According to this theory, some guardians may inadvertently reinforce their dogs’ attention‐seeking behaviours, leading to over‐dependence [34].

The study recorded a significant good training score of 70.8%, consistent with García Artica’s [25] findings of 80.2% but higher than Torres [26], who only found 56.7%. This trait is not considered a behavioural problem but rather a temperament characteristic influenced by prior training experiences, age and handling [35]. Such results are expected in dogs participating in sports, as their motivation and enthusiasm make them more receptive to commands and foster effective collaboration with their handlers, enhancing obedience levels. For other behavioural issues, a rate of 22.5% was observed in our pilot study. These other behaviours are considered problematic, and a low score may be due to the dogs’ suitability for agility sports, characterised by focus and adaptability in structured environments. Nevertheless, monitoring and managing excessive vocalisations and ensuring adequate energy levels for training remain important considerations.

Most dogs were Border Collies (90%), a preference probably due to the Border Collie’s attributes, such as its energy and trainability, which are as important as its physical appearance [36]. Regarding sex, 50% corresponded to neutered female or male dogs, which aligns with data showing that only 61.2% of dogs in Chile are sterilised [37]. None of the surveyed dogs was younger than the minimum age required to start agility sports. This is consistent with Kennel Agility Chile, which specifies that 18 months is the minimum age for dogs to participate in Grade I competitions. Our preliminary results show that this is also happening for agility dogs where most people felt their dogs as member of their family. Interestingly, 30% of guardians did not classify their agility dogs as family members, despite displaying 100% compliance with veterinary care and welfare standards. This result presents an intriguing research question rather than an indicator of compromised welfare. Viewing a dog primarily through a functional or sporting lens (utilitarian or working‐animal bond) rather than an anthropomorphic humanistic lens (family member) may reflect distinct handler attachment styles. Future studies should investigate whether this functional perception influences handler expectations and training methodologies (e.g., strict task‐oriented focus vs. companion bonding) and whether it buffers against or predisposes dogs to specific behavioural disorders such as separation anxiety. Main limitations of the study were the small number of animals and lack of a representative dog group comparison. Future studies should be conducted with a larger number of dogs to corroborate the findings. Also, although we compared our preliminary results with other studies in working dogs or the general population of dogs using C‐BARQ, it would have been good to have a nonagility group of dogs. However, we believe this is a good starting point for future studies involving more agility and nonagility dogs (working or nonworking dogs) in different countries.

## Funding

This research received no funding.

## Conflicts of Interest

The authors declare no conflicts of interest.

## Data Availability

The data that support the findings of this study are available from the corresponding author upon reasonable request.

## References

[1] Svartberg K. and Forkman B. , Personality Traits in the Domestic Dog (*Canis familiaris*), Applied Animal Behaviour Science. (2002) 79, no. 2, 133–155, 10.1016/S0168-1591(02)00121-1.

[2] Cavalli C. M. , Carballo F. , and Bentosela M. , Gazing Behavior During Problem Solving Tasks in Domestic Dogs. A Critical Review, Dog Behavior. (2018) 4, 23–44, 10.4454/DB.V4I3.68.

[3] Pastore C. , Pirrone F. , Balzarotti F. , Faustini M. , Pierantoni L. , and Albertini M. , Evaluation of Physiological and Behavioral Stress-Dependent Parameters in Agility Dogs, Journal of Veterinary Behavior. (2011) 6, no. 3, 188–194, 10.1016/J.JVEB.2011.01.001.

[4] Hecht J. and Horowitz A. , Introduction to Dog Behavior, Animal Behavior for Shelter Veterinarians and Staff, 2015, 3–30, 10.1002/9781119421313.ch1.

[5] Buttner A. P. , Thompson B. , Strasser R. , and Santo J. , Evidence for a Synchronization of Hormonal States Between Humans and Dogs During Competition, Physiology and Behavior. (2015) 147, 54–62, 10.1016/J.PHYSBEH.2015.04.010. 25862521

[6] Kuźniewska E. and Strychalski J. , Perceptions of Domestic Dogs’ (*Canis familiaris*) Manifestations of Joy, Shame and Stress Based on Photographs, Dog Behavior. (2021) 7, 21–34, 10.4454/DB.V7I3.142.

[7] Marshall-Pescini S. , Passalacqua C. , Barnard S. , Valsecchi P. , and Prato-Previde E. , Agility and Search and Rescue Training Differently Affects Pet Dogs’ Behaviour in Socio-Cognitive Tasks, Behavioural Processes. (2009) 81, no. 3, 416–422, 10.1016/J.BEPROC.2009.03.015. 19520242

[8] Ramey C. , Pechette Markley A. , Olson R. et al., AGILE: Agility Innovations Leveraging Electronics, ACM International Conference Proceeding Series. (2023) 1–9, 10.1145/3637882.3637889.

[9] Niewiadomska M. , Forms of Activities With a Dog as Modern Types of Physical Recreation, Central European Journal of Sport Sciences and Medicine. (2018) 23, 53–58, 10.18276/CEJ.2018.3-05.

[10] Chapagain D. , Virányi Z. , Wallis L. , Huber L. , Serra J. , and Range F. , Aging of Attentiveness in Border Collies and Other Pet Dog Breeds: The Protective Benefits of Lifelong Training, Frontiers in Aging Neuroscience. (2017) 9, 10.3389/fnagi.2017.00100. PMC5397477 28473766

[11] Ákos Z. , Beck R. , Nagy M. , Vicsek T. , and Kubinyi E. , Leadership and Path Characteristics During Walks Are Linked to Dominance Order and Individual Traits in Dogs, PLoS Computational Biology. (2014) 10, no. 1, 10.1371/JOURNAL.PCBI.1003446. PMC3900374 24465200

[12] Salonen M. , Mikkola S. , Hakanen E. , Sulkama S. , Puurunen J. , and Lohi H. , Reliability and Validity of a Dog Personality and Unwanted Behavior Survey, Animals. (2021) 11, no. 5, 10.3390/ANI11051234. PMC8147106 33923262

[13] Mirkó E. , Dóka A. , and Miklósi Á. , Association Between Subjective Rating and Behaviour Coding and the Role of Experience in Making Video Assessments on the Personality of the Domestic Dog (*Canis familiaris*), Applied Animal Behaviour Science. (2013) 149, no. 1-4, 45–54, 10.1016/J.APPLANIM.2013.10.003.

[14] Meegahapola L. , Constantinides M. , Radivojevic Z. , Li H. , Quercia D. , and Eggleston M. S. , Quantified Canine: Inferring Dog Personality From Wearables, *Conference on Human Factors in Computing Systems—Proceedings*, 2023.

[15] Hsu Y. and Serpell J. A. , Development and Validation of a Questionnaire for Measuring Behavior and Temperament Traits in Pet Dogs, Journal of the American Veterinary Medical Association. (2003) 223, no. 9, 1293–1300, 10.2460/JAVMA.2003.223.1293. 14621216

[16] Foyer P. , Bjällerhag N. , Wilsson E. , and Jensen P. , Behaviour and Experiences of Dogs During the First Year of Life Predict the Outcome in a Later Temperament Test, Applied Animal Behaviour Science. (2014) 155, 93–100, 10.1016/J.APPLANIM.2014.03.006.

[17] Hunt R. L. , England G. C. W. , Asher L. , Whiteside H. , and Harvey N. D. , Concurrent and Predictive Criterion Validity of a Puppy Behaviour Questionnaire for Predicting Training Outcome in Juvenile Guide Dogs, Animals. (2020) 10, no. 12, 10.3390/ANI10122382. PMC7763496 33322634

[18] Tonoike A. , Nagasawa M. , Mogi K. , Serpell J. A. , Ohtsuki H. , and Kikusui T. , Comparison of Owner-Reported Behavioral Characteristics Among Genetically Clustered Breeds of Dog (*Canis familiaris*), Scientific Reports. (2015) 5, no. 1 5, 1–11, 10.1038/srep17710. PMC4683527 26680442

[19] Sanders A. R. , Bhongir N. , vonHoldt B. , and Pellegrini M. , Association of DNA Methylation With Energy and Fear-Related Behaviors in Canines, Frontiers in Psychology. (2022) 13, 10.3389/FPSYG.2022.1025494. PMC9794564 36591016

[20] Scandurra A. , Mastellone V. , Pero M. E. et al., Effects of a Nutritional Supplement (DìRelaxTM) on Anxiety in Dogs in a Randomized Control Trial Design, Animals. (2022) 12, no. 4, 10.3390/ANI12040435. PMC8868118 35203143

[21] Wauthier L. M. , Scottish Society for the Prevention of Cruelty to Animals (Scottish SPCA) , and Williams J. M. , Using the Mini C-BARQ to Investigate the Effects of Puppy Farming on Dog Behaviour, Applied Animal Behaviour Science. (2018) 206, 75–86, 10.1016/J.APPLANIM.2018.05.024.

[22] Wilkins V. , Evans J. , Park C. , Fitzpatrick A. L. , Creevy K. E. , and Ruple A. , Validation of the Shortened Version of the Canine Behavioral Assessment and Research Questionnaire (C-BARQ) Using Participants From the Dog Aging Project, PLoS One. (2024) 19, no. 4, 10.1371/JOURNAL.PONE.0299973. PMC11008875 38603705

[23] Oikonomidis I. L. , Tsouloufi T. K. , Kritsepi‐Konstantinou M. , and Soubasis N. , The Effect of Age and Sex on Glycated Hemoglobin in Dogs, Journal of Veterinary Diagnostic Investigation. (2021) 34, no. 2, 331–333, 10.1177/10406387211065046. PMC8921804 34931567

[24] Duffy D. L. , Kruger K. A. , and Serpell J. A. , Evaluation of a Behavioral Assessment Tool for Dogs Relinquished to Shelters, Preventive Veterinary Medicine. (2014) 117, no. 3-4, 601–609, 10.1016/J.PREVETMED.2014.10.003. 25457136

[25] García Artica C. , Determinación de la Prevalencia de los Problemas de Comportamiento Observados en los Perros que Asisten a una Clínica de Animales Menores en Base a la Percepción de los Dueños, 2019, Universidad Científica del sur, Lima, Peru, Undergraduate thesis.

[26] Torres S. , Determinación y Caracterización de Problemas de Comportamiento en Perros de la Ciudad de Valdivia Mediante la Aplicación del Cuestionario C-BARQ (Canine Behavior Assessment and Reseach Questionnaire), 2009, Universidad Austral de Chile, Undergradute thesis.

[27] González-Ramírez M. T. , Quezada-Berumen L. , and Landero-Hernández R. , Assessment of Canine Behaviors Using C-BARQ in a Sample From Northern Mexico, Journal of Veterinary Behavior. (2017) 20, 52–58, 10.1016/j.jveb.2017.03.007.

[28] Luna Gavilanez C. J. , Determinacion de las Poblaciones de Riesgo Frente a la Agresividad de Perros Pitbull y Rottweiler a Partir del año de Edad en Tres Clinicas Veterinarias de la Ciudad de Latacunga, 2011, Universidad Técnica de Cotopaxi.

[29] DiMichele J. K. , Markley A. P. , Shoben A. B. , and Kieves N. R. , Evaluation of Variability in Performance and Paw Placement Patterns by Dogs Completing the Dog Walk Obstacle in an Agility Competition, PLoS One. (2024) 19, no. 3, 10.1371/journal.pone.0299592. PMC10923472 38457394

[30] Väätäjä H. , Majaranta P. , Cardó A. V. et al., The Interplay Between Affect, Dog’s Physical Activity and Dog–Owner Relationship, Frontiers in Veterinary Science. (2021) 8, 10.3389/fvets.2021.673407. PMC8695727 34957271

[31] Stellato A. C. , Flint H. E. , Widowski T. M. , Serpell J. A. , and Niel L. , Assessment of Fear-Related Behaviours Displayed by Companion Dogs (*Canis familiaris*) in Response to Social and Non-Social Stimuli, Applied Animal Behaviour Science. (2017) 188, 84–90, 10.1016/J.APPLANIM.2016.12.007.

[32] Conley M. J. , Fisher A. D. , and Hemsworth P. H. , Effects of Human Contact and Toys on the Fear Responses to Humans of Shelter-Housed Dogs, Applied Animal Behaviour Science. (2014) 156, 62–69, 10.1016/J.APPLANIM.2014.03.008.

[33] Willen R. M. , Schiml P. A. , and Hennessy M. B. , Enrichment Centered on Human Interaction Moderates Fear-Induced Aggression and Increases Positive Expectancy in Fearful Shelter Dogs, Applied Animal Behaviour Science. (2019) 217, 57–62, 10.1016/J.APPLANIM.2019.05.001.

[34] McCrave E. A. , Diagnostic Criteria for Separation Anxiety in the Dog, Veterinary Clinics of North America: Small Animal Practice. (1991) 21, no. 2, 247–255, 10.1016/S0195-5616(91)50030-9. 2053248

[35] Serpell J. A. and Hsu Y. , Effects of Breed, Sex, and Neuter Status on Trainability in Dogs, Anthrozoös. (2005) 18, no. 3, 196–207, 10.2752/089279305785594135.

[36] Rossi V. , El Border Collie, 2018, De Vechi Ediciones.

[37] Subsecretaría de Desarrollo Regional y Administrativo , Boletín Técnico Estimación de la Población Canina y Felina del País y Diagnóstico de la Tenencia Responsable, 2022.

